# Supplementary material for: HIVprotI: an integrated web based platform for prediction and design of HIV proteins inhibitors
Source: J Cheminform. 2018 Mar 9;10:12. doi: 10.1186/s13321-018-0266-y (PMC5845081; doi:10.1186/s13321-018-0266-y)
Supplement: Supplementary file 2 — Additional file 2. Source code of HIVProtI web server. [file 13321_2018_266_MOESM2_ESM.zip › HIVprotI_Source-code/hivprotI/weka-3-6-6/doc/weka/classifiers/evaluation/CostCurve.html]

CostCurve


---


|  |  |  |
| --- | --- | --- |
| **Overview** \| **Package** \| **Class** \| **Tree** \| **Deprecated** \| **Index** \| **Help** \| **Weka's home** | |  |
| **PREV CLASS**   **NEXT CLASS** | **FRAMES**    **NO FRAMES**     **All Classes** |  |
| SUMMARY: NESTED \| FIELD \| CONSTR \| METHOD | DETAIL: FIELD \| CONSTR \| METHOD |  |


---


## weka.classifiers.evaluation Class CostCurve

```
java.lang.Object
  weka.classifiers.evaluation.CostCurve
```

**All Implemented Interfaces:**: RevisionHandler

---

``` public class CostCurve extends java.lang.Object implements RevisionHandler ```

Generates points illustrating probablity cost tradeoffs that can be
obtained by varying the threshold value between classes. For example,
the typical threshold value of 0.5 means the predicted probability of
"positive" must be higher than 0.5 for the instance to be predicted as
"positive".

**Version:**
:   $Revision: 1.9 $

**Author:**
:   Mark Hall (mhall@cs.waikato.ac.nz)

---

| **Field Summary** | |
| --- | --- |
| `static java.lang.String` | `NORM_EXPECTED_COST_NAME`             attribute name: Normalized Expected Cost |
| `static java.lang.String` | `PROB_COST_FUNC_NAME`             attribute name: Probability Cost Function |
| `static java.lang.String` | `RELATION_NAME`             The name of the relation used in cost curve datasets |
| `static java.lang.String` | `THRESHOLD_NAME`             attribute name: Threshold |


**Constructor Summary**

`CostCurve()`


| **Method Summary** | |
| --- | --- |
| `Instances` | `getCurve(FastVector predictions)`             Calculates the performance stats for the default class and return results as a set of Instances. |
| `Instances` | `getCurve(FastVector predictions, int classIndex)`             Calculates the performance stats for the desired class and return results as a set of Instances. |
| `java.lang.String` | `getRevision()`             Returns the revision string. |
| `static void` | `main(java.lang.String[] args)`             Tests the CostCurve generation from the command line. |

**Methods inherited from class java.lang.Object**

`equals, getClass, hashCode, notify, notifyAll, toString, wait, wait, wait`

**Field Detail**

### RELATION\_NAME

```
public static final java.lang.String RELATION_NAME
```

:   The name of the relation used in cost curve datasets

    **See Also:**: Constant Field Values

---


### PROB\_COST\_FUNC\_NAME

```
public static final java.lang.String PROB_COST_FUNC_NAME
```

:   attribute name: Probability Cost Function

    **See Also:**: Constant Field Values

---


### NORM\_EXPECTED\_COST\_NAME

```
public static final java.lang.String NORM_EXPECTED_COST_NAME
```

:   attribute name: Normalized Expected Cost

    **See Also:**: Constant Field Values

---


### THRESHOLD\_NAME

```
public static final java.lang.String THRESHOLD_NAME
```

:   attribute name: Threshold

    **See Also:**: Constant Field Values


**Constructor Detail**

### CostCurve

```
public CostCurve()
```


**Method Detail**

### getCurve

```
public Instances getCurve(FastVector predictions)
```

:   Calculates the performance stats for the default class and return
    results as a set of Instances. The
    structure of these Instances is as follows:

    - **Probability Cost Function** - **Normalized Expected Cost**- **Threshold** contains the probability threshold that gives
          rise to the previous performance values.

    :   **Parameters:**: `predictions` - the predictions to base the curve on **Returns:**: datapoints as a set of instances, null if no predictions have been made. **See Also:**: `TwoClassStats`

---


### getCurve

```
public Instances getCurve(FastVector predictions,
                          int classIndex)
```

:   Calculates the performance stats for the desired class and return
    results as a set of Instances.

    :   **Parameters:**: `predictions` - the predictions to base the curve on: `classIndex` - index of the class of interest. **Returns:**: datapoints as a set of instances.

---


### getRevision

```
public java.lang.String getRevision()
```

:   Returns the revision string.

    :   **Specified by:**: `getRevision` in interface `RevisionHandler`
    :   **Returns:**: the revision

---


### main

```
public static void main(java.lang.String[] args)
```

:   Tests the CostCurve generation from the command line.
    The classifier is currently hardcoded. Pipe in an arff file.

    :   **Parameters:**: `args` - currently ignored


---


|  |  |  |
| --- | --- | --- |
| **Overview** \| **Package** \| **Class** \| **Tree** \| **Deprecated** \| **Index** \| **Help** \| **Weka's home** | |  |
| **PREV CLASS**   **NEXT CLASS** | **FRAMES**    **NO FRAMES**     **All Classes** |  |
| SUMMARY: NESTED \| FIELD \| CONSTR \| METHOD | DETAIL: FIELD \| CONSTR \| METHOD |  |


---
